# Prediction of a Cell-Class-Specific Mouse Mesoconnectome Using Gene Expression Data

**DOI:** 10.1007/s12021-020-09471-x

**Published:** 2020-05-24

**Authors:** Nestor Timonidis, Rembrandt Bakker, Paul Tiesinga

**Affiliations:** 1 Neuroinformatics Department, Donders Centre for Neuroscience, Radboud University Nijmegen, Heyendaalseweg 135, 6525 AJ Nijmegen, the Netherlands; 2 Institute of Neuroscience and Medicine (INM-6) and Institute for Advanced Simulation (IAS-6) and JARA BRAIN Institute I, Jülich Research Centre, Wilhelm-Johnen-Strasse, 52425 Jülich, Germany

**Keywords:** Spatial gene co-expression, Connectomics, Machine learning, Predictive models, Mouse brain, Axonal projection, Gene expression, Gene ontology enrichment analysis, Ridge regression, Dictionary learning, Sparse coding, ROC analysis, Cellularly resolved connectome

## Abstract

Reconstructing brain connectivity at sufficient resolution for computational models designed to study the biophysical mechanisms underlying cognitive processes is extremely challenging. For such a purpose, a mesoconnectome that includes laminar and cell-class specificity would be a major step forward. We analyzed the ability of gene expression patterns to predict cell-class and layer-specific projection patterns and assessed the functional annotations of the most predictive groups of genes. To achieve our goal we used publicly available volumetric gene expression and connectivity data and we trained computational models to learn and predict cell-class and layer-specific axonal projections using gene expression data. Predictions were done in two ways, namely predicting projection strengths using the expression of individual genes and using the co-expression of genes organized in spatial modules, as well as predicting binary forms of projection. For predicting the strength of projections, we found that ridge (L2-regularized) regression had the highest cross-validated accuracy with a median *r*^2^ score of 0.54 which corresponded for binarized predictions to a median area under the ROC value of 0.89. Next, we identified 200 spatial gene modules using a dictionary learning and sparse coding approach. We found that these modules yielded predictions of comparable accuracy, with a median *r*^2^ score of 0.51. Finally, a gene ontology enrichment analysis of the most predictive gene groups resulted in significant annotations related to postsynaptic function. Taken together, we have demonstrated a prediction workflow that can be used to perform multimodal data integration to improve the accuracy of the predicted mesoconnectome and support other neuroscience use cases.

**Electronic supplementary material:**

The online version of this article (10.1007/s12021-020-09471-x) contains supplementary material, which is available to authorized users.

## Introduction

A wiring diagram of the brain (connectome) is a necessary step for advancing modern neuroscience for two reasons. First, it assists computational neuroscience by providing biologically plausible constraints on brain models and simulations (Choi and Mihalas 2019). Second, it bridges the gap between experimental data and computational models by providing frameworks exposing its graph-theoretical structure and other properties (Sanz-Leon et al. 2013; Ritter et al. 2013; Woodman et al. 2014). Examples of connectome based projects are the Blue Brain project or the Virtual Brain project that aim to create large-scale models of the rodent or human brain (Markram 2006; Markram et al. 2011; Sanz Leon et al. 2013).

The meso-scale description of the connectome (mesoconnectome) is defined at the level of anatomically distinct sub-areas within each brain region and is typically described by the use of tract-tracing invasive techniques in animal studies, or post mortem dissections in human studies (Kötter 2007; Sporns et al. 2005; Highley et al. 1999; Lanciego and Wouterlood 2011). The whole brain coverage provided by these techniques and the ability to delineate layer-specific sub-areas make the mesoconnectome neither too coarse grained nor too spatially limited and thus suitable for developing computational models of structural brain connectivity (Oh et al. 2014; Knox et al. 2018; Betzel et al. 2015a; Betzel et al. 2015b).

It is difficult with tract-tracing techniques to get good whole brain coverage and they are time consuming (Sporns 2011). As an alternative to classical neuroanatomy, gene-expression-based approaches have been used to describe the connectome for a number of reasons (Fornito et al. 2019). First, it is possible to infer connectivity information from gene expression based on the premise that postsynaptic structures have specific protein profiles and that neurons connected through synapses have highly correlated gene expression patterns (Roy et al. 2018; Sperry 1963; Fornito et al. 2019). Second, the recent advances in sequencing have resulted in gene expression data being high throughput, relatively cheap and easy to obtain (Shendure and Ji 2008).

These advantages have led to various studies linking genomic information with structural brain connectivity using computational approaches (Baruch et al. 2008; Kaufman et al. 2006; French and Pavlidis 2011; French et al. 2011; Wolf et al. 2011). In recent studies, a link has been established between gene expression and the mouse mesoconnectome by building predictive models and associating gene co-expression with network topology and structure (Rubinov et al. 2015; Fulcher and Fornito 2014; Ji et al. 2014), resulting in computational frameworks for the mouse mesoconnectome.

Despite the aforementioned advances, research in the field still faces a number of limitations. An example is the lack of brain-wide descriptions of important cytoarchitectonic features of the connectome, such as the number of axonal fibers and the density of axonal arbor endings, for neuronal populations categorized by their projection patterns (also referred to as projection cell-classes) or by their transcriptomic profiles (transcriptomic cell-types) (Harris et al. 2018; Tasic et al. 2018; Tasic 2018). These features could be used for quantifying long-range connections between neuronal populations which could then lead to a cell-type-specific mouse mesoconnectome.

Descriptions with that level of resolution have been provided at the local microcircuit level of the mouse brain but are limited to specific brain areas such as the primary visual cortex (Lee et al. 2016). Moreover, features such as axonal fiber and arbor endings can not be extracted from models describing the connectome as a binary network of present or absent axonal projections between brain areas (Ji et al. 2014; Fulcher and Fornito 2014).

In this work we measure the amount of information about axonal projection patterns present in gene expression patterns of the mouse brain and we associate the findings with factors related to the functional annotations of genes. For that purpose we have used publicly available volumetric gene expression and connectivity data from the Allen Institute for Brain Science. In order to bridge the gap between coarse-level mesoscale predictions and cell-type-specific predictions, we have trained computational models to learn and predict cell-class and layer-specific axonal projections using gene expression. Predictions are made in two ways, namely predicting projection volumes using the expression patterns of individual genes and using the co-expression of genes organized in spatial modules, as well as predicting binary forms of projection. For analyzing the functional annotations of the most predictive gene groups, we use gene ontology enrichment analysis.

The primary scientific contribution of this paper is to advance the level of prediction from binary — is a pair of areas connected — to continuous — how strongly are the areas connected, which is more relevant for computational models and overcomes the limitations of binary network models mentioned above. While binary predictions are part of our analysis, binarization of projection volume is achieved with a data-driven approach that does not rely on arbitrary thresholding. We further show that these approaches not only work for the older wild-type data set (Oh et al. 2014) but also for individual cre-lines (Harris et al. 2019), which allows for the integration of cell-class-specific projection patterns in the mesoconnectome for use in models. Moreover, the use of spatial genes modules for predicting axonal projections and gene ontology analysis establishes a relationship between functional gene groups and the mesoconnectome and is a step towards integrating transcriptomic cell-types in connectome analysis.

Based on that framework, we have built a predictive workflow that is focused on integrating gene expression and structural connectivity data related to the mouse brain which are available from a number of repositories. In this paper we describe the predictive workflow (Methods) and we quantify and compare the performance of various ways of making predictions (Results). This includes predictions based on the full gene expression data (continuous and binary mode), and based on gene expression organized in spatial gene modules. For the most predictive genes we perform a gene ontology analysis. An open-source implementation of the various use-cases is described in the Supplemental Methods section.

## Methods

**Fig. 1:**
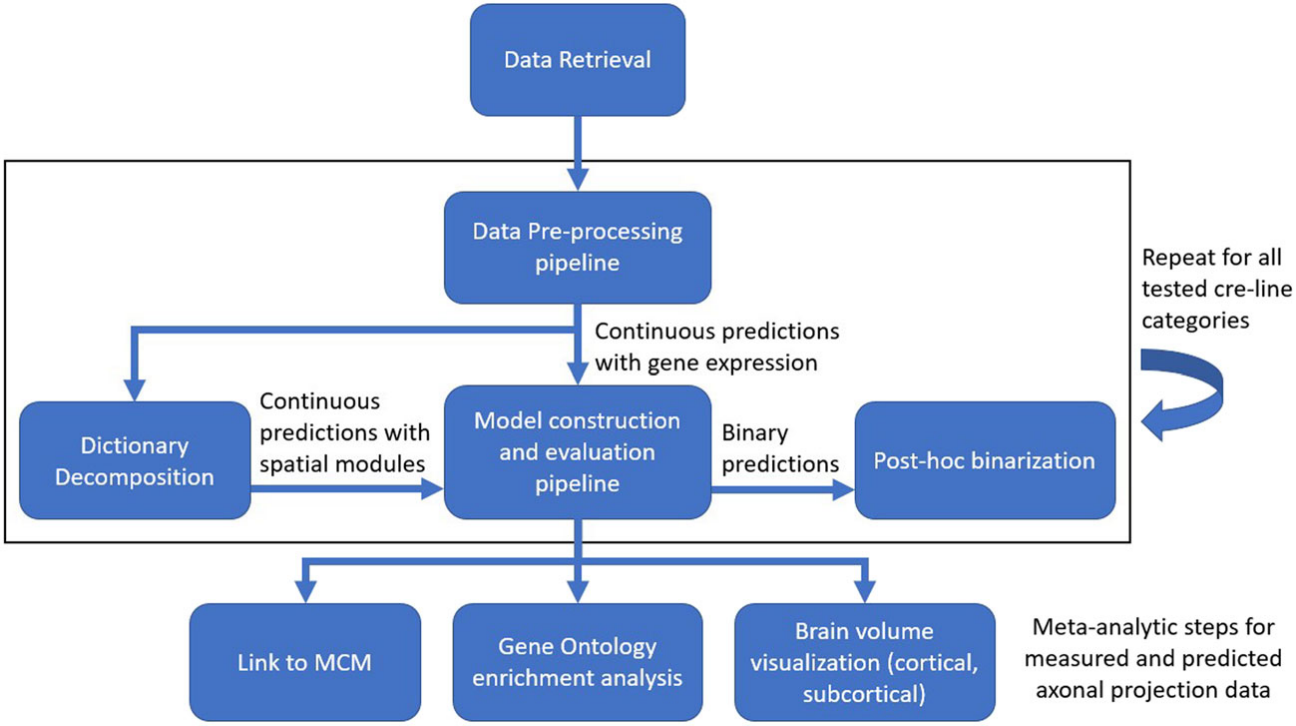
Flowchart describing the various steps of the predictive workflow

We developed a predictive workflow to measure the amount of information about axonal projection patterns present in gene expression patterns of the mouse brain and to associate it with factors related to the functional annotations of genes (Fig. 1). Here we describe what data we used and how these were pre-processed as well as the various steps of the analysis. See Section S1.1 for information related to the software packages used to implement our methods.

### Materials

#### Allen Mouse Brain Atlas

The gene expression data were obtained from the Allen Mouse Brain Atlas (AMBA) dataset of the Allen Institute for Brain Science (Table 1), (Lein et al. 2007). The *in situ hybridization* (ISH) technique was used to quantify the spatial expression patterns of $\sim $20.000 genes in the brains of male C57BL/6J (wild-type) mice which were 56-days-old (P56). ISH constitutes a high throughput approach for quantifying expression energies of multiple genes in multiple spatial locations with up to 1 *μm* resolution (Lein et al. 2007; Amann and Fuchs 2008).

**Table 1:** Hyperlinks for websites, tool descriptions and format descriptions related to our analysis. See main text for details

Allen Institute	https://alleninstitute.org/
MCC documentation	https://allensdk.readthedocs.io/en/latest/connectivity.html
CCF v3.0	http://help.brain-map.org/display/mouseconnectivity/Documentation
MCC use case	https://alleninstitute.github.io/AllenSDK/_static/examples/nb/mouse_connectivity.html
MCM tool	https://mouse-connectivity-models.readthedocs.io/en/latest/
NIfTI files	https://nifti.nimh.nih.gov/
JSON files	https://en.wikipedia.org/wiki/JSON
SBA Composer	https://scalablebrainatlas.incf.org/composer-dev/?template=ABA_v3
Bioconductor software	http://bioconductor.org/packages/release/data/annotation/html/org.Mm.eg.db.html
Scikit-learn library	https://scikit-learn.org/stable/
Repository of our Code on the HBP Collaboratory	https://collab.humanbrainproject.eu/#/collab/8650/nav/65518
Repository of our Code on Github	https://github.com/ntimonid/Connectomic-Composition-Predictor-CCP-
Neuroexpresso Tool	https://github.com/PavlidisLab/markerGeneProfile
Cortical flatmap templates	https://download.alleninstitute.org/informatics-archive/current-release/mouse_ccf/cortical_coordinates/ccf_2017/

In the study that created the AMBA dataset (Lein et al. 2007), in situ hybridization was used together with fluorescence microscopy in order to visualize the gene expression energy. The result of this analysis was a set of sagittal and coronal brain slice images containing the expression energy of $\sim $20000 and $\sim $3300 individual genes respectively (Lein et al. 2007). The coronal slices were selected for our analysis because their in plane resolution was higher.

#### Allen Mouse Brain Connectivity Atlas

The axonal projection data were obtained from the Allen Mouse Brain Connectivity Atlas (AMBCA) dataset. These data were based on the anterograde tract-tracing technique that was used to quantify the strength of axonal projections within the brains of P56 wild-type and transgenic cre-line mice using two-photon microscopy and producing brain slice images reaching up to 1 *μm* resolution (Oh et al. 2014; Harris et al. 2018) (see Supplementary File 1 for more details on primary brain regions).

In anterograde tract-tracing, viruses are injected to a source brain area where they produce fluorescent molecules that reach target brain areas by being transported along the axons and reaching the axonal terminals. In Oh et al. (2014) (referred to as the wild-type experiments) they used a recombinant adeno-associated virus (rAAV) expressing enhanced green fluorescent protein (EGFP), whereas in Harris et al. (2018) (referred to as the cre-line experiments) they utilized a cre-recombinase-dependent rAAV virus expressing a synaptophysin-EGFP fusion that labels presynaptic terminals (sypEGFP) and an EGFP expressed in the cytoplasm of infected neurons. The main difference is that in the wild-type experiments, most neurons at the injection location were labeled, whereas in the cre-line experiments, only neurons belonging to specific cell-classes were labeled.

From the 49 major transgenic cre-lines tested in Harris et al. (2018), we selected the 14 most frequently used ones (899 out of 1080 experiments), and thus obtained axonal projections from cortical areas with different laminar labeling profiles and emerging from different cell-classes (Harris et al. 2014, 2018). There were 3 cell-classes, namely corticothalamic (CT), intratelencephalic (IT) and pyramidal tract (PT), that were defined based on the long range projection properties of the excitatory neurons labeled in the corresponding cre-line. There were 4 laminar profiles that were defined based on the cortical layers having the most excitatory neurons labeled in the corresponding cre-line (L2/3, L4, L5 and L6, see full table in Supplementary File 2). The cre-lines together with the 498 available wild-type experiments constituted the 15 tract-tracing categories processed in this study comprising 1397 experiments in total.

### Allen Pre-Processing Pipeline

The brain slice images were processed using the informatics processing pipeline of the Allen Institute for Brain Science (Table 1). Specifically, they were registered and aligned in the same reference space according to the latest version of the Allen mouse brain atlas, Common Coordinate Framework version 3 (CCF v3.0) (Table 1).

The last step in the informatics processing pipeline was the unionization process during which the volume of both data modalities was averaged over anatomically distinct brain areas. With unionized/regionalized we mean that all voxels of a volumetric data set within the same brain region are processed together. When obtaining the unionized data we selected target brain areas of the right hemisphere, since the tracing experiments have been performed in the right hemisphere and they mostly target the same hemisphere (ipsilateral projections).

Proper measures had to be selected for voxel unionization of both data modalities. The gene expression data were unionized using the *expression energy* measure, which for a given gene is defined as the sum of expression intensity of pixels divided by the sum of all pixels in a particular brain area.

The projection data were unionized using the *normalized projection volume*, which for a given tracing experiment is defined as the sum of detected pixels of projection to a particular brain area divided by the number of pixels in that area and further normalized by the sum of all pixels covered by the corresponding injection. Both measures were estimated over all genes or tracing experiments respectively and over all target brain areas (see Supplementary File 1). As a result, 2D arrays were created whose rows corresponded to target brain areas and columns corresponded to tracing experiments or genes depending on the modality (Oh et al. 2014; Ji et al. 2014).

### Data Retrieval

In our predictive workflow we used three sources of neuroanatomical data, namely gene expression, wild-type tracing experiments and cre-line tracing experiments, and the latter two were downloaded with the mouse connectivity cache (MCC) API (Table 1).

We packaged and pre-processed the data as follows (Fig. S2). First, experiments corresponding to gene expression or tract tracing were downloaded from the Allen Institute. Second, the unionized gene expression experiments were packed in a 2-dimensional array where rows correspond to target anatomically-defined brain areas and columns correspond to individual genes. Third, for each wild-type and cre-line tracing experiment, a matrix was created with rows corresponding to target brain areas and columns corresponding to individual injections associated with source brain areas. Finally, all tracing-related matrices were assembled into one aggregate data structure together with tracing-related metadata such as the cell-class and layer specificity of injections, acronyms of source areas and injection coordinates (see Supplementary Files 1 and 2).

### Pre-Processing Pipeline

We searched for not-a-number (NaN) values in the gene expression and axonal tracing datasets and removed them based on their frequency of occurrence (Fig. S4). We removed 610 out of 1038 anatomical brain areas defined by CCF v3.0 because they had a large fraction (> 80 *%*) of NaNs in either the gene expression or the axonal tracing datasets. The remaining NaN values in the Gene Expression dataset were imputed by taking the median value of the corresponding gene for all non-NaN brain areas (Fig. S7).

For the tracing data, a sampling-based imputation approach was followed (Fig. S7). To ensure that zero values would also have a chance of being used for imputing missing values, we stratified projection values per column (that is per tracing experiment) into zero and non-zero values. For each missing value present in a column, one of these groups was chosen with a probability proportional to its fraction in the non-missing data and from the chosen group a random value was drawn to be used for the imputation.

We subsequently rescaled both data modalities, first by applying a cube root transformation in order to decrease the skewness of their distributions (Fig. S6), followed by a z-score transformation in order to ensure that the regression-based predictive models were trained faster (Friedman et al. 2009). The z-score was obtained by subtracting the mean across areas and normalizing with the corresponding standard deviation (Fig. S7).

### Dictionary Decomposition

The gene expression data were decomposed into transcriptional networks represented by spatial gene modules and coefficients. The Dictionary Learning and Sparse Coding (DLSC) method was used for decomposition, in which a data array is being represented by a linear combination of sparse but non-orthogonal modules or dictionaries and their coefficients (Mairal et al. 2010; Li et al. 2017). This technique allows us to visually inspect various gene co-expression patterns in the mouse brain and reduces redundancy, since genes belonging to the same co-expression network have a putatively similar function across the brain (Langfelder and Horvath 2008). In DLSC both the coefficients and dictionaries are obtained by minimizing the deviation from the data under a *l*_1_ constraint on the coefficients (atoms) and non-negativity constraints on the elements of both the dictionaries and the coefficients:

1 $$ \begin{array}{@{}rcl@{}} (D,a)&=&\text{argmin}\quad \frac{1}{2}||X - Da||_{2}^{2},\\ || a||_{1} &\leq& \lambda, \quad ||a|| > 0,\quad D_{ij} > 0, \quad \forall i,j\in\mathbb{N} \end{array} $$

In our analysis the data array (X) corresponded to the gene expression matrix (brain areas × genes), atoms (a) corresponded to the coefficients of individual genes to each module (modules × genes) and dictionaries (D) corresponded to the spatial gene modules of the mouse brain (brain areas × modules). We set the *λ* hyperparameter (*l*_1_ constraint) to 1.0 and we applied two different DLSC-based factorizations to the data.

First, we exclusively used the ISH data and chose the number of dictionaries by training models to predict tract-tracing experiments with a different number of spatial modules, and then selecting a model with a high *r*^2^ score (see Fig. 4). We selected a set of 200 modules despite being second in performance (the median *r*^2^ is 0.51 for 200 modules and 0.52 for 300 modules), since the set of 300 modules was considered to be too large and their difference was considered to be an effect of variability (both interquartile ranges are 0.19 as shown by the vertical lines in Fig. 4, panel C) These modules accounted on average for 10% of variability across genes and had an average spatial footprint covering 88% of the brain areas, which were labeled as *unconstrained spatial modules*.

Second, in order to formulate cell-type-specific densities in a similar fashion to previous studies (Grange et al. 2014), we obtained the expression patterns of 74 cell-types from single-cell RNA sequencing data available at the neuroexpresso repository (Tasic et al. 2016; Mancarci et al. 2017) (see Table 1) and we selected 2154 common genes between the single-cell and the ISH data, which resulted in a 2154 × 74 array of cell-type-specific gene expression. Consequently, we constrained the DLSC model by setting the atoms to be equal to the cell-type-specific data, re-used the ISH data as input to the model and selected 74 modules in order to match the number of cell-types.

Therefore, the resulting matrices comprised of 428 areas × 200 *unconstrained spatial modules* and 428 areas × 74 *constrained spatial modules*, respectively, which was a significant reduction in dimensionality compared to the 428 × 3318 ISH gene expression matrix.

### Model Construction Pipeline

A separate prediction model was built for each cre-line or wild-type category as follows. First, the gene expression data and the modules of gene co-expression were trained with either the Random Forest or Ridge Regression method (Tikhonov and Arsenin 1977; Dietterich 2000; Breiman 2001; Friedman et al. 2009). Subsequently, model performance was validated with nested 3-fold cross-validation (Kohavi 1995; Varma and Simon 2006) and quantified by the *r*^2^ score between the measured and predicted projection patterns. For more information regarding the implementation of these techniques, see Sections S1.2–S1.4. The *r*^2^ score is defined as the fraction of total variance of the measured patterns that can be explained by the predicted ones (Dodge 2008):

2 $$ r^{2} = 1 - \frac{{\sum}_{i}{(y_{i} - f_{i})^{2}}}{{\sum}_{i}{(y_{i} - \tilde{y})^{2}}},  $$

here the index i represents brain regions, y represents a ground truth vector, $\tilde {y}$ represents its mean and f represents the predicted version of the vector. Finally, the predicted projection patterns with their optimal hyperparameter set were extracted as model outputs.

As additional post-hoc analyses, the predictions were converted to binary, using our post-hoc binarization approach, and a gene ontology enrichment analysis was applied to the most predictive gene groups for finding significant annotations related to neuronal and synaptic functions (Fawcett 2006; Rivals et al. 2007; Rice 2007). For more information about the post-hoc analysis steps, see Sections S1.5 and S1.6.

## Results

We downloaded gene expression and axonal projection data from the Allen Brain Atlas and Allen Mouse Brain Connectivity Atlas repositories, which were spatially registered and aligned to the latest version of the Allen mouse brain atlas, Common Coordinate Framework version 3 (CCF v3.0). The axonal projection data were represented by unionized and normalized projection volumes derived from anterograde tract tracing experiments that were comprised of 1397 distinct injection sites, of which the majority (n = 498) was from wild-type subjects and the remainder were from 14 different cre-lines of transgenic animals. The gene expression data were represented by unionized expression energies across brain areas. Both data modalities were pre-processed to remove brain areas with poor quality data, impute missing values and rescale values to an appropriate range for fitting (see Methods).

### Prediction of Continuous Projection Volume Based on Gene Expression Patterns

**Fig. 2:**
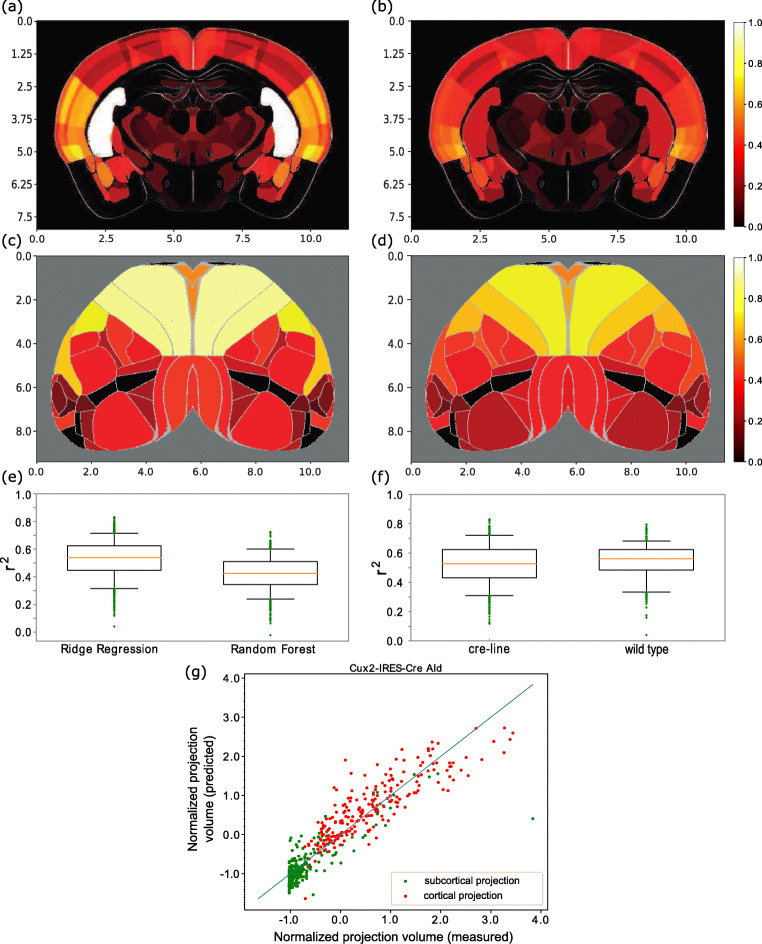
Brain volume visualizations and prediction performance statistics related to continuous models trained with different methods to predict tract tracing datasets. **a**-**d** Subcortical and cortical visualizations for a Cux2-IRES-Cre-line tracing experiment which labeled IT cells in layers 2/3 and was injected to AId (agranular insular area, dorsal part). **a**, **c**: measured values. **b**, **d**: predictions from gene expression patterns. The subcortical projection patterns were visualized using coronal slices of the normalized projection volume, whereas the cortical projection patterns were projected onto a flatmap and their values were averaged over all cortical layers. The scale for both axes is in milimeters, the colormap used ranges from black through red, orange, and yellow, to white and corresponds to normalized projection volume ranging from 0 to 1, while the intensity of each plot has been normalized by its maximum value. Cortical areas such as the retrosplenial area dorsal part (RSPd), anteromedial visual area (VISam), trunk of primary somatosensory area (SSP-tr) and posterior auditory area (AUDpo) exhibit highly similar normalized projection volume between their measured and predicted versions, while subcortical similarities are not as strong as the cortical ones. **e** Prediction performance comparison of Ridge Regression (left) with Random Forest (right) based models over all tract-tracing experiments. y-axis: *r*^2^ scores. The red line is the median, the box encloses the interquartile range and the green dots are outliers which comprised 0.7 *%* of the injections for Ridge Regression and 2*%* for the Random Forest. **f** Comparison of wild-type based models with cre-line based models trained using Ridge Regression. x-axis: tract-tracing category. y-axis: *r*^2^ values. **g** Prediction performance scatter plot for the Cux2-IRES-Cre-line expe riment. The *r*^2^ score for this experiment was 0.826, which was the highest score across all tracing experiments. x-axis: measured data. y-axis: predicted data. Green points correspond to subcortical projections, red points to cortical ones and the solid line is the diagonal, for which predicted values are equal to the measured ones

We explored various fitting procedures for predicting the normalized projection volume from the gene expression data. The two supervised learning methods used for fitting the data were Random Forest and Ridge Regression, while the performance was measured using the *r*^2^ score which represents the fraction of total variance accounted for by the model. Across all injection sites, irrespective of subject type, Ridge Regression based predictions yielded a median *r*^2^ of 0.54 with an interquartile range (iqr) of 0.178. Random forest based predictions yielded a median *r*^2^ score of 0.42, which was lower than the one for the Ridge Regression based predictions (Fig. 2). We show an example in Fig. 2, which demonstrates the outcome of predictions using nested 3-fold cross-validation with Ridge Regression.

Variation in performance was analyzed across experiments of different tract-tracing categories. When the performance was partitioned according to transgenic cre-line and wild-type, the performance of wild-type was approximately in the center of the range for transgenic animals. As the number of injections in each transgenic cre-line was much lower than available wild-type data (n = 12 to 125 for transgenic versus n = 498 for wild-type) this variation can be most likely attributed to experimental variability, rather than the specific properties of a transgenic line. Our statistical tests, using similarly sized subsets of wild-type data, indicated that the difference was not statistically significant (p = 0.004 for 100 random permutations per cre-line, 14000 permutations in total, with the same distribution in set size as the cre-lines).

Predictions of projection patterns with the Ridge Regression-based models trained on gene expression data were significant. The Ridge Regression models trained with actual gene expression patterns outperformed in every case surrogate models, which were created by randomly distributing the expression intensity of each gene across areas (for three representative cases see Fig. 2). This process was repeated 25 times for each cre-line and wild-type tracing experiment. The predictive models that were trained with the surrogate data, also referred to as surrogate models, had a median *r*^2^ score of -0.005 (iqr = 0.007) over all tracing experiments.

All of the Ridge Regression models outperformed the null models, that were incorporated into the analysis as an additional control (Fig. 2). The null models predicted unseen projection patterns by averaging values of the seen ones and thus did not account for variability across brain areas. A model was considered inaccurate when it was outperformed by those null models. The null models had a median *r*^2^ score of -0.003 (iqr = 0.005) over all tracing experiments.

Predictions with low *r*^2^ values can be expected when multiple projection patterns need to be predicted simultaneously. Specifically, the models were trained to fit simultaneously multiple tracing experiments belonging to a particular tracing category (i.e. wild-type mice) with the same set of 3318 genes and the same hyperparameter set. In our data, 10 out of 1397 tracing experiments (0.7%) had a value in the range [0 - 0.2] for Ridge Regression based models, while the equivalent percentage for Random Forest based models was 40 out of 1397 (2.8%).

Nevertheless, performance of models with a high *r*^2^ score can be appreciated when the predicted projection patterns are visually compared with the measured ones in the form of computed brain slices and cortical flatmaps (for an example see Fig. 2).

### Binary Predictions

**Fig. 3:**
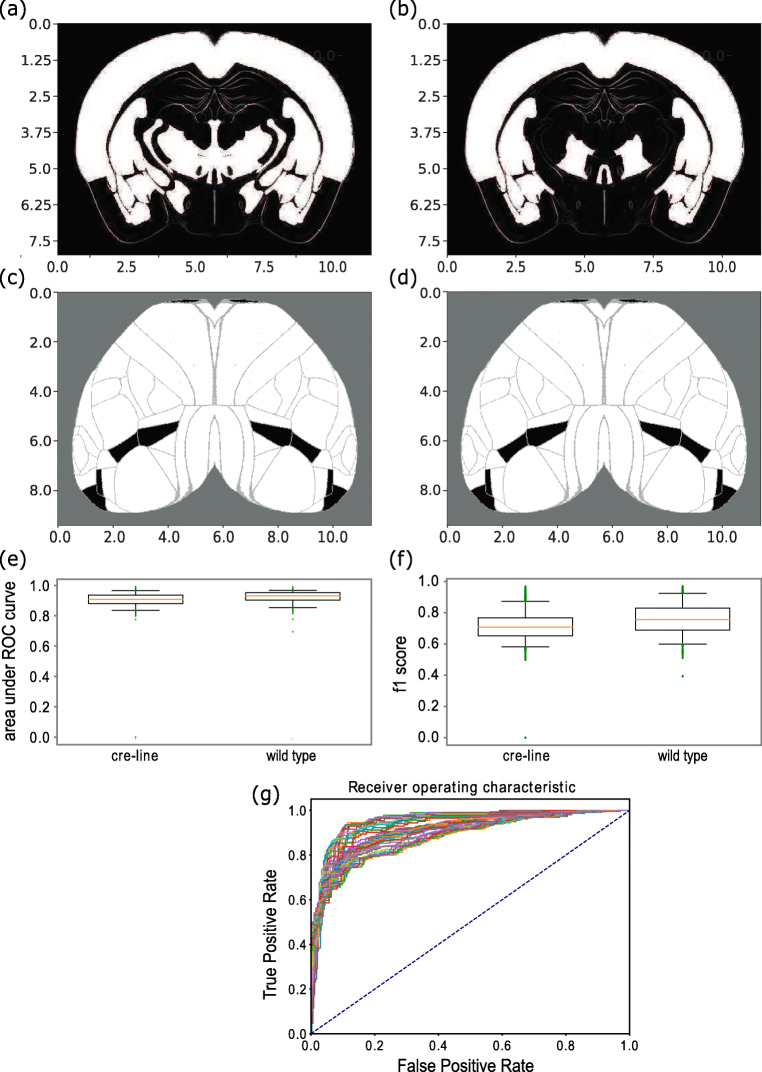
Brain volume visualizations and prediction performance statistics related to binary models trained with Ridge Regression to predict tract tracing datasets. **a**-**d** Subcortical and cortical visualizations for the binarized form of a Cux2-IRES-Cre line tracing experiment injected to the AId area (agranular insular area, dorsal part). **a**, **b**: measured values. **b**, **d**: predictions from gene expression patterns. The subcortical projection patterns were visualized using coronal slices of the normalized projection volume **a**, **b**, whereas the cortical projection patterns **c**, **d**, were projected onto a flatmap. The scale for both axes is in milimeters. White denotes the value 1 (connections present), and black denotes the absence of a projection. **e**-**f** Comparison of wild-type based models with cre-line based models. x-axis: tract-tracing category. y-axis: auROC (e) and f1-score **f** values. The red line is the median, the box encloses the interquartile range and the green dots are outliers. In both boxplots, two green dots with a value of 0 for both auROC and f1-score correspond to two Ntsr1-Cre_GN220 line tracing experiments injected at the lateral visual area (VISl) and expressed in L6 CT neurons, for which 100*%* of their values were thresholded to 0 during binarization due to the median and standard deviation of their normalized projection volumes being too low (1.46^− 10^ median and 0.01 std for the first experiment and 8.063^− 11^ median and 0.07 std for the second experiment). **g** Multi-ROC curve between measured and predicted projection patterns of the Nr5a1-Cre line tracing experiment. The ROC curves correspond to multiple curves induced by applying multiple external thresholds to the measured data

Previous studies have used a binarized version of the mesoconnectome to test the accuracy of their predictive models. In order to compare our performance to these models, we developed an approach to make binary predictions as well (see Section S1.5, Fig. 3). The accuracy of these predictions was quantified using an ROC analysis with as outcome the area under the ROC curve (auROC).

The median auROC value over all 1397 tracing experiments was 0.89 (median iqr = 0.08) (Fig. 3). Moreover, performance for wild-type data matched that of the state of the art in binary projection predictions of wild-type experiments with gene expression data, such as in Ji et al. (2014), where a 0.93 auROC was obtained. The auROC values for all wild-type tracing experiments also had a median of 0.93 (iqr = 0.05). Similar values for cre-lines were obtained, which had not been subject to this type of analysis before (Harris et al. 2018). For instance, the auROC values for Tlx3-Cre_PL56 line tracing experiments, labeling IT cells in layers 2-6, had a median of 0.94 (iqr = 0.03) (Fig. 3).

Visualization of measured and predicted results, in the form of cortical flatmaps and coronal slices, allows for assessing the quality of predictions in spatial context. An example is the Cux2-IRES-Cre line tracing experiment injected to AId area (Fig. 3), which had an auROC value of 0.98 for binary prediction.

The increased performance of the models on binary predictions compared to continuous ones (Fig. 3) was due to reduced information about the projection patterns, which can therefore be more easily captured by the gene expression data. However, the resulting connectivity descriptions are on a very coarse-grained level which made the continuous ones more suitable for analytic purposes.

### Gene Module Analysis

We used the Dictionary Learning and Sparse Coding technique (DLSC) with the intention of identifying functional gene groups with a similar spatial distribution related to cell-type-specific densities. To test the DLSC technique under different constraints, we used the ISH data exclusively and provided 200 *unconstrained spatial modules*, followed by 74 *constrained spatial modules* by cell-type-specific gene expression data obtained from the neuroexpresso repository (Tasic et al. 2016; Mancarci et al. 2017).

**Fig. 4:**
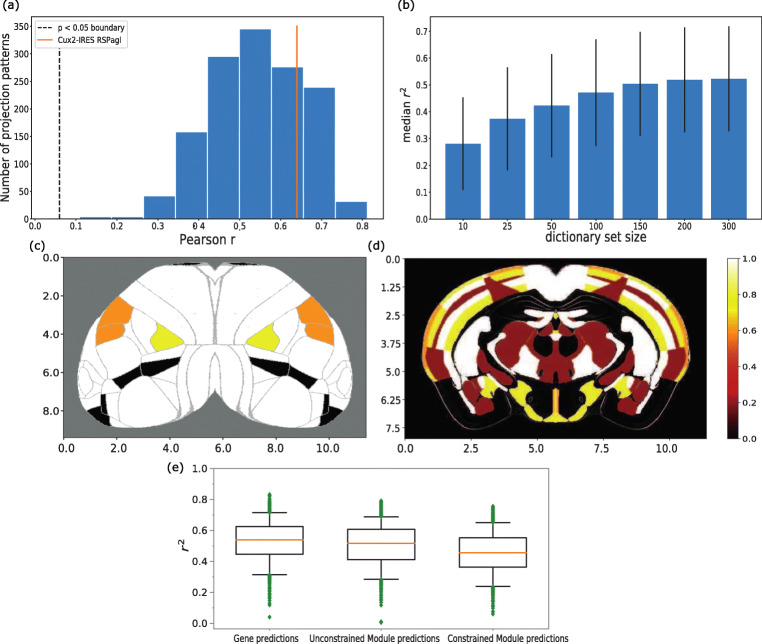
Statistics of the prediction performance of models trained using spatial gene modules and correlations between the modules and axonal projection volumes. **a** Histogram of Pearson correlation coefficients (r) between all 1397 tracing experiments and their predicted forms. The prediction of each experiment was achieved with its 3 best correlated modules as determined by Pearson r. The first vertical line from the left represents the point at which all correlations left to it are no longer statistically significant (p > 0.05). The second vertical line from the left corresponds to the Pearson r of 0.64 between a Cux2-IRES-Cre line tracing experiment injected to the retrosplenial area, lateral agranular part (RSPagl), and modules 9, 70 and 88, which is greater than the mean r of 0.54. A dense distribution of correlations in the range 0.4 - 0.7 indicates that multiple spatial modules correlate with axonal projection patterns. **b** Predictive performance of module-based models with different dictionary set sizes, trained with Ridge Regression. x-axis: dictionary set size. y-axis: median *r*^2^ score over all tract-tracing experiments. The vertical lines represent the interquartile range across the dictionary sets. The highest peak is for 300 modules with an *r*^2^ score of 0.52. (c-d) Cortical (**c**) and subcortical (**d**) visualization of a spatial footprint related to the Cux2-IRES-Cre line RSPagl tracing experiment. The spatial footprint represents the overlap that exists between the RPSagl experiment and modules 9, 70 and 88 with a Pearson r of 0.51, 0.52 and 0.45 respectively. Each non-zero value across brain areas is replaced by 1.0 if it was present in all three modules and the projection pattern, 0.8 if present in two modules and the pattern, 0.6 if present in one module and the pattern, 0.4 if present in the pattern and absent in all modules and 0.2 if present in the modules but absent in the pattern. The subcortical projection pattern was visualized using coronal slices of the normalized projection volume, whereas the cortical projection was projected onto a flatmap and its values were averaged over all cortical layers. The scale for both axes is in milimeters. There is a strong presence of white (1.0), yellow (0.8) and orange (0.6) colors, suggesting a strong overlap between the experiment and the modules and which is also reflected by a *r*^2^ score of 0.4 when the three modules are used for predicting the experiment. **e** Comparison of predictive accuracy between models trained using spatial modules and models trained using full gene expression data. The method used is Ridge Regression. x-axis: gene expression based models (left), spatial module-based models (middle) and module-based models constrained with single-cell RNA sequencing data (right). y-axis: *r*^2^ scores

**Fig. 5:**
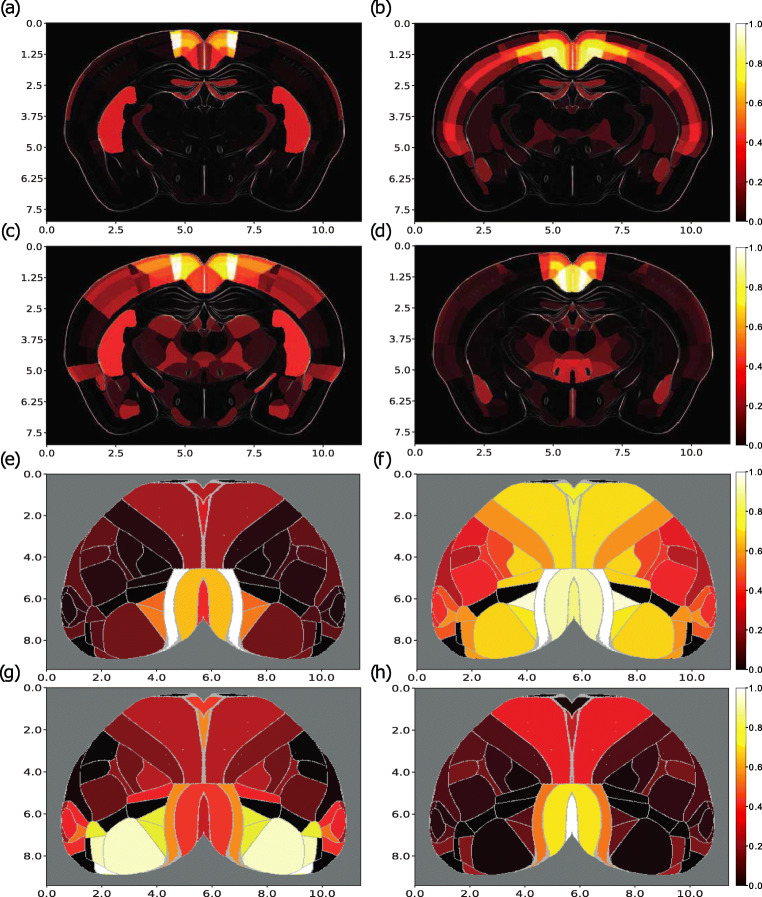
Subcortical (**a**) and cortical (**e**) visualizations for the Cux2-IRES-Cre line RSPagl tracing experiment compared to visualizations of spatial gene modules 9 (**b**,**f**), 70 (**c**,**g**) and 88 (**d**,**h**). The subcortical projection or module patterns were visualized using coronal slices of the normalized projection volume (**a**) or module expression (**c-d**), whereas the cortical projection or module patterns (**c**-**h**) were projected onto a flatmap and their values were averaged over all cortical layers. The scale for both axes is in milimeters, the colormap used ranges from black through red, orange, and yellow, to white and corresponds to normalized projection volume or module expression ranging from 0 to 1, while the intensity of each plot has been normalized by its maximum value

In order to examine the predictive capabilities of the spatial modules, the prediction process was repeated with models trained on the modules instead of genes. We considered an example tracing experiment for which the unconstrained-module-based predictive model had the highest *r*^2^ score of 0.79. The tracing experiment was generated by a Cux2-IRES-Cre line injection in the retrosplenial area, lateral agranular part (RSPagl). We looked for unconstrained modules with the highest similarity with the projection pattern, as quantified using the Pearson correlation coefficient (r). We selected three modules, labeled as 9, 70 and 88, with a Pearson r of 0.51, 0.52 and 0.45 respectively. Each of these modules were non-zero in a mostly non-overlapping group of brain areas, which together cover a part of the experimental projection pattern (see Figs. 4 and 5). We analyzed the contribution of their spatial footprint in each area separately, as indicated in Fig. 4, which highlighted a large overlap between the experiment and the modules in cortical areas. Subcortical areas did not have such a strong coverage as cortical ones, which might be the reason why predictive performance was not higher in terms of the *r*^2^ score.

Subsequently, we calculated the Pearson r between the RSPagl experiment and its prediction by the three modules. We found that this prediction yielded an *r*^2^ score of 0.4 and a Pearson r value of 0.64, which was higher than the median Pearson r of 0.54 over all tracing experiments. Therefore, these modules were important components to the total prediction, whereas they provided a less accurate prediction as stand-alone predictors (see Figs. 4 and 5).

This finding suggests that multiple spatial modules might be needed to reproduce projection density patterns from the mouse cortex. For the predictive models trained and tested with unconstrained spatial modules over all tracing experiments, the median and maximum *r*^2^ scores were 0.51 and 0.79 (iqr = 0.19), respectively, while the corresponding median *r*^2^ score of the constrained models was 0.45 (iqr = 0.19). Therefore the unconstrained module results were slightly lower on average than the corresponding ones for the gene predictions but of higher quality than the constrained ones (Fig. 4), while a biclustering analysis between the two types of modules did not result in meaningful biclusters and suggested that there is little relationship between them (Fig. S9). For testing the significance of module-based predictions, surrogate models were built as explained in Section 2 and trained with spatial modules instead of genes. All models trained for the 1397 tracing experiments had higher *r*^2^ values than the respective surrogate ones, as indicated by a number of examples in Fig. 4.

A gene ontology analysis was applied to the unconstrained spatial modules and the models using different tract-tracing experiments in order to identify significant annotations related to synaptic and neuronal function in the mouse brain (Rivals et al. 2007). For each tracing experiment, we included the most predictive genes whose model coefficients exceeded the 99^*th*^ percentile, while for each spatial module, we included all genes having a non-zero coefficient.

**Fig. 6:**
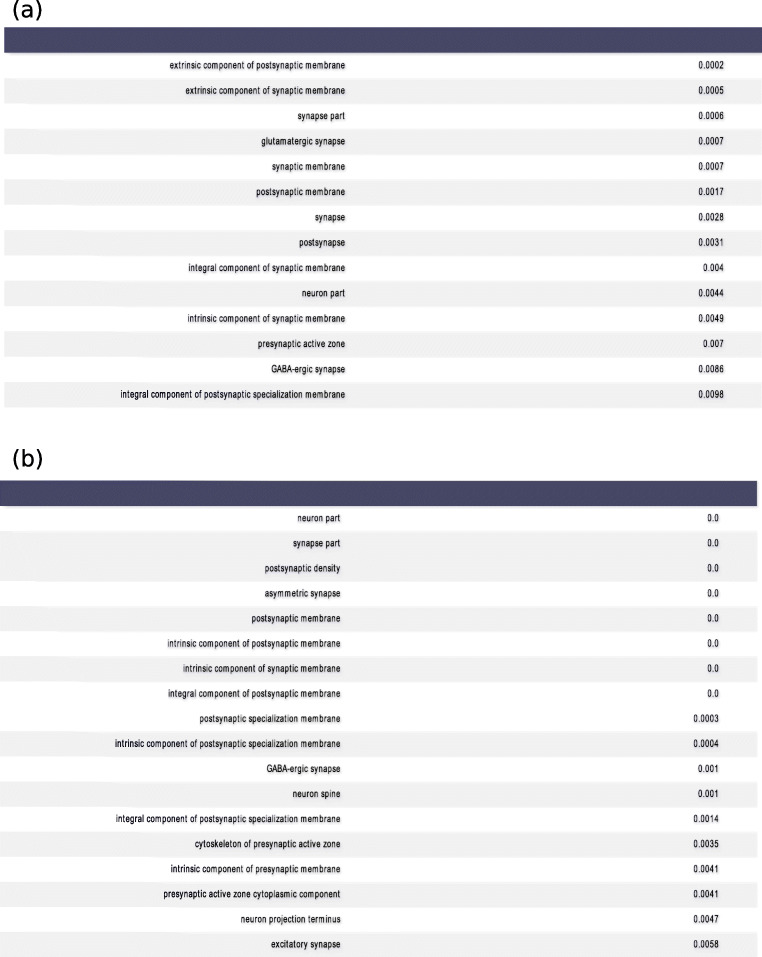
An enrichment analysis reveals annotations of neurons and synapses for a spatial module and a tracing experiment. **a** Significant annotations for a Cux2-IRES-Cre line experiment injected to the RSPagl area. **b** Significant annotations for the constrained spatial gene module 9. The hypergeometric test has been used for finding significant associations between genes and annotations (p ≤ 0.05)

The percentage of modules and tracing experiments having at least one significant annotation was 100% and 98%, respectively, while a tracing experiment was associated with 12 annotations on average (median) and a module was associated with 39 annotations on average (see Fig. 6 for indicative examples). As a generalization of this observation, the percentage of modules and tracing experiments having at least one annotation related to postsynaptic function was 100% and 70%, respectively. Hence, the presence of postsynaptic function annotations was another common denominator between a substantial number of tracing experiments and spatial modules, in addition to strong correlations and predictive capability.

## Discussion

In this study we built a predictive workflow that was focused on integrating gene expression and structural connectivity data related to the mouse brain (Fig. 1). We measured the amount of information about axonal projection patterns present in gene expression patterns of the mouse brain and we associated the findings with factors related to the functional annotations of genes. We predicted projection volumes using expressions of individual genes (continuous predictions), and we predicted binary forms of projection using individual gene expression (binary predictions). Subsequently we predicted projection volumes using spatial modules of genes that were defined based on the DLSC method. For the continuous gene expression predictions, we obtained a median *r*^2^ of 0.54 over 1397 tract-tracing experiments, which when converted to binary predictions corresponded to similar performance to previous studies (Ji et al. 2014). Regarding the spatial gene module predictions, we obtained median *r*^2^ scores of 0.51 and 0.45 for the unconstrained and constrained approaches respectively. In gene ontology enrichment analysis, a substantial number of the groups were found to be associated with annotations related to postsynaptic function. In the following we will put the performance of the different methods in the context of previous studies, interpret our findings, suggest potential future work and discuss strengths, limitations and other applications of our workflow.

The results of our study are consistent with the findings from the (Ji et al. 2014) study, specifically since our binary approach yielded a similar performance with a median 93% auROC value on wild-type data. In contrast to this study however, we did not rely on arbitrary thresholds for binarizing each tracing experiment to attain a 50% connectivity. Instead, we provided a data-driven estimation of the most optimal threshold value. In addition, we extended their analysis by including cre-line data that had not been subjected to such an analysis before.

When including both cre-line and wild-type data, we found a median auROC value of 0.89 across all 1397 tracing experiments. The increased performance of the models on binary predictions compared to continuous predictions is presumably due to the reduction of projection pattern related information which can therefore be more easily captured by the gene expression data (Fig. 3). However, binary connectivity descriptions do not inform the modeler about the strength of a projection. Hence, the continuous predictions are more suitable for analytic purposes. For that reason, we provided richer predictions of the mouse mesoconnectome by incorporating continuous patterns to our analysis (Fig. 2 for continuous predictions and Fig. 3 for binary ones).

Overall, our Ridge Regression models provided significant predictions, since they outperformed in every case the surrogate and the null models. This implies that gene expression contains information related to axonal projection patterns in the mouse brain. Regarding the variability of predictions, our statistical tests indicated that the difference in performance between cre-line and wild-type tracing experiments, quantified as *r*^2^ score, was not statistically significant (p = 0.004 for 14000 random permutations). A possible explanation is that both wild-type and cre-line projection patterns fall within the range of predictions that can be covered by the gene expression data. Irrespective of explanation, the results show that the gene expression data contain enough information to also account for the more specific cre-line projection patterns.

The Ridge Regression models trained with spatial gene co-expression modules rather than expressions of individual genes, also outperformed corresponding surrogate and null models (Fig. 4). However, we found that such predictions were slightly less accurate on average than the gene expression based ones. Despite that, significant predictions of such models and strong correlations between axonal projections and spatial modules suggest that measured patterns of individual genes contain more variability unrelated to projection patterns than patterns of a limited number of modules.

When comparing constrained modules with the unconstrained ones, we observed dissimilar patterns and a reduced performance for the constrained one when predicting tracing experiments. Such results suggest a lack of direct relation between spatial modules created exclusively by ISH data and modules that were constrained by single cell RNA sequencing data. A possible explanation is that distinct predictive modules were mixed when including all genes differentially expressed in the 74 cell-types, which suggests that better performance could be reached when selecting a subset from amongst them.

Regarding gene ontology enrichment analysis, a substantial number of tracing experiments (70%) and all unconstrained spatial modules (100%) were statistically associated with postsynaptic function. This may suggest that a potential causal link between axonal projections and gene expression in the mouse brain could be gene co-expression modules with a postsynaptic function and specific spatial footprints. This suggestion is consistent with the findings of Roy et al. (2018), according to which presynaptic and postsynaptic locations have a particular protein profile. These profiles are partially reflected in gene expression data by locally expressed genes at axonal release sites (Glock et al. 2017; Cajigas et al. 2012; Holt and Schuman 2013). Nevertheless, the putative causal links are far from being clear and will thus require further work.

A strength of this study was the inclusion of layer and cell-class-specific patterns by including cre-line data to our analysis. To the best of our knowledge, this is the first study that predicts brain-wide and cell-class-specific projection patterns from gene expression data. Another advantage of this study was that it went beyond solely providing a predictive workflow, and it focused on discovering links between the two data modalities by analyzing the spatial organizations of genes with the dictionary learning and sparse coding technique (Li et al. 2017) and with gene ontology enrichment analysis (Rivals et al. 2007).

We acknowledge some limitations. First, the use of cre-lines and their predictions does not fully provide cell-type-specific axonal projections since cre-lines label neuronal populations at the source and not at the target level. Furthermore, the labeled cell-classes of IT, PT and CT neurons do not fully overlap with transcriptomic cell-types and do not capture cellular diversity in the mouse cortex as accurately as the latter do (Tasic et al. 2016, 2018; Zeisel et al. 2015).

Another issue concerning the use of cre-lines is their layer specificity. In Zeisel et al. (2015), they identified 7 layer-specific subclasses of pyramidal cells located in the primary somatosensory cortex. Genes serving as markers for cre-lines such as Cux2 and Rorb were found to be expressed in multiple of these subclasses. This showed that these cre-lines can label multiple layers instead of a single one over all cortical areas and that they have been associated with their most frequently labeled layer. Despite these issues, the cre-line inclusion was the first step in providing mesoscale projection patterns with variability on the level of cortical areas, layers and cell-classes, which added depth in modeling and analysis of the mesoconnectome.

Regarding limitations of the predictive models, 0.7% of Ridge Regression based models had an r^2^ score close to zero. This could be attributed to hyperparameters being optimized over all tracing experiments belonging to one cre-line or the wild-type category rather than for each experiment (injection) separately. Furthermore, examples such as the Ntsr1-Cre_GN220 line tracing experiment at the VISl area (see Fig. 3) indicated the presence of experiments with sparse brain coverage of projections. Therefore, it can be expected that performance will be reduced when multiple projection patterns need to be predicted simultaneously.

Another explanation could be that including the genetic information of target areas without its relation to source areas has limited capacity in predicting projection patterns. Nevertheless, according to Fulcher and Fornito (2014), correlated gene expression patterns were shown to be directly linked with the large-scale topology of the mouse mesoconnectome. Furthermore, in Bleakley et al. (2007) they used the support vector machine algorithm with kernels that coupled the feature vectors of nodes for inferring the edges of metabolic and protein-interaction networks. As a recommendation for future work, we can adapt this strategy to couple source and target based gene expression patterns and infer their corresponding axonal projections. Another limitation is that unionization of data leads to information loss, not-a-number values and projection bias because of diversity in sizes of source brain areas. For that reason we will focus our future analyses on the volumetric gene expression and axonal projections data, as to avoid such issues and provide a finer grained predictive workflow.

Furthermore, Ridge Regression and Random Forest based models provided significant predictions of axonal projections from gene expression data, but they are not capable of explicitly modeling the joint distribution between the two data modalities. Such explicit modeling could be advantageous in the case of training models to predict cellularly resolved projections since data that could serve as training labels, such as single-neuron axonal reconstruction data, are still limited (Economo et al. 2019; Winnubst et al. 2019). Future directions might include incorporating generative probabilistic models, since models such as the infinite relational model have been successful in capturing the distributions of various connectomes such as the C.elegans connectome and the mouse retina microcircuit (Jonas and Kording 2015; Ambrosen et al. 2013; Hinne et al. 2014; Hinne et al. 2017; Betzel and Bassett 2017).

Whole brain cellularly resolved connections have yet to be described. The capability of our models to provide information for a more faithful reconstruction of the connectome at this resolution will depend on two factors. The first factor will be the ability to incorporate new advances in neuroanatomy and translational neuroscience, such as single-cell RNA sequencing and light sheet fluorescence microscopy (Tasic 2018; Corsetti et al. 2019; Rolnick and Dyer 2019).

The second factor will be the ability to mine at a higher spatial resolution from already tested data modalities such as in-situ hybridization based gene expression data. For this factor we will need to adapt additional computational tools for use in our workflow. One potential tool is spatial point process analysis, which has successfully been used to extract spatially distributed counts of cells and synapses from modalities such as Nissl-stained brain images (LaGrow et al. 2018; Anton-Sanchez et al. 2014).

Despite their limitations, our predictive models can be tested in new use-cases and for different resolutions as long as genetic data are available and registered to the Allen CCF v3.0 (see Section S2). Taken together, we have demonstrated a predictive workflow that can further be used to perform multimodal data integration to improve the accuracy of the predicted mouse mesoconnectome using gene expression data and support other neuroscience use cases.

## Information Sharing Statement

A number of workflow-related use-cases have been designed and tested in the form of Jupyter Notebooks and have been published online with their descriptions at the HBP Collaboratory and at Github. See Main Table 1 for links to the notebooks and to public repositories of the tools and modules mentioned here.

## Electronic supplementary material

Below is the link to the electronic supplementary material.

(CSV 1.36 KB)

(CSV 354 bytes)

(PDF 3.29 MB)
