# Supplementary material for: Prediction of a Cell-Class-Specific Mouse Mesoconnectome Using Gene Expression Data
Source: Neuroinformatics. 2020 May 24;18(4):611–26. doi: 10.1007/s12021-020-09471-x (PMC7498447; doi:10.1007/s12021-020-09471-x)
Supplement: Supplementary file 3 — (PDF 3.29 MB) [file 12021_2020_9471_MOESM3_ESM.pdf]

Neuroinformatics manuscript No.
(will be inserted by the editor)

Prediction of a cell-class-specific mouse mesoconnectome using gene expression data

Nestor Timonidis · Rembrandt Bakker · Paul Tiesinga

Received: date / Accepted: date

1 Supplementary Methods

1.1 Code

The main programming language used in this analysis was Python 2.7, with the R-3.2 programming language having a supplementary role in gene ontology analysis. The number of workflow-related use-cases have been designed and tested in the form of Jupyter Notebooks and have been published online at the HBP Collaboratory and at Github. The predictive models were implemented with the use of the scikit-learn library. Various tools from the NumPy and SciPy libraries were selected for the numerical calculations and the univariate statistical analyses, respectively. The visualizations were performed with the Matplotlib 2D plotting library of Python. We utilized the Allen Software Development Kit (SDK) for downloading and processing the unionized data and the Mouse Connectivity Models (MCM) tool for linking our workflow with the regionalized connectivity models. For performing gene ontology enrichment analysis, we employed the rpy2 library which enabled us to link our Python scripts with the R-implemented hyperGTest function from the GOHyperGParams-

Nestor Timonidis
[1]Neuroinformatics department, Donders Centre for Neuroscience, Radboud University Nijmegen, Heyendaalseweg 135, 6525 AJ Nijmegen, the Netherlands
Tel.: +31-649552777
E-mail: n.timonidis@donders.ru.nl

Rembrandt Bakker
[1]Neuroinformatics department, Donders Centre for Neuroscience, Radboud University Nijmegen, Heyendaalseweg 135, 6525 AJ Nijmegen, the Netherlands
[2]Inst. of Neuroscience and Medicine (INM-6) and Inst. for Advanced Simulation (IAS-6) and JARA BRAIN Inst. I, Jülich Research Centre, Wilhelm-Johnen-Strasse, 52425 Jülich, Germany.

Paul Tiesinga
[1]Neuroinformatics department, Donders Centre for Neuroscience, Radboud University Nijmegen, Heyendaalseweg 135, 6525 AJ Nijmegen, the Netherlands

class package provided by Bioconductor. See Main table 1 for links to repositories of the tools and modules mentioned here.

1.2 Ridge Regression

Ridge Regression (also referred to as Tikhonov regularization) is a form of penalized linear regression commonly used in supervised machine learning and regression statistics (Tikhonov and Arsenin, 1977; Friedman et al., 2009). Classical linear regression fits a 2-dimensional array X to a vector y by estimating a coefficient vector w that minimizes the residuals between the actual y and the predicted $\hat{y}$ estimated as: $\hat{y}$ = Xw - b, where b is an intercept term.
The ordinary least squares method is used for optimizing the coefficient vector (Friedman et al., 2009):

$$\hat{w} = \text{argmin}||y - Xw - b||_2^2 \quad (1)$$

In cases of high dimensional data, where the number of features is greater than the number of samples, the dataset exhibits high variance and thus noise which hinders the generalization performance of the trained model. Ridge regression deals with the problem by constraining the size of the coefficients (Friedman et al., 2009). This is done by adding the l_2 norm of the coefficients, multiplied by a shrinkage hyperparameter λ, to the objective function:

$$\hat{w} = \underset{w \in R}{\text{argmin}}(||y - Xw - b||_2^2 + \lambda ||w||_2^2) \quad (2)$$

The greater the value of λ the greater the shrinkage of the coefficients towards zero (Friedman et al., 2009). In our analysis we utilized Ridge Regression in order to fit predictive models using gene expression data to learn projection patterns of the tract-tracing data and predict unseen patterns. For each predictive model, the most optimal λ value was chosen among multiple values in the range [10^{-3} - 10^5]. The hyperparameter selection strategy is shown in section S 1.4.

1.3 Random Forest Regressor

Random Forest Regressor is an ensemble method for performing regression tasks (Dietterich, 2000; Breiman, 2001). The basic premise for ensemble methods is that averaging reduces variance. In Random Forest the ensemble is comprised of multiple Decision Trees (Breiman, 2001), with the number of Trees being a hyperparameter of the method.
In our analysis we utilized Random Forest Regressor with 200 Decision Trees as an ensemble alternative to Ridge Regression in order to investigate differences in the predictive performance between different methods. Moreover according to literature, it constitutes a robust approach against data overfitting that occurs when the training data error is significantly lower than the testing data error (Breiman, 2001).

1.4 Internal Model Validation

For an internal evaluation of our predictive models, a technique called nested k-fold cross-validation was applied to the dataset, which is an alternative to the classical k-fold cross-validation technique (Kohavi, 1995; Bishop, 2006). Classical cross-validation is biased since both model performance evaluation and hyperparameter optimization can only be tested simultaneously on the same folds, and there is no independent set to test both factors separately. Nested cross-validation deals with the issue by nesting each training fold with internal training and validation folds and applying k-fold cross-validation to internal folds for selecting the most optimal hyperparameter set (Varma and Simon, 2006). Since each external testing fold tests a model whose hyperparameter set has been selected from other folds, the aforementioned bias is avoided (figure S 8).
Furthermore, the overall stability of the trained models can be tested by comparing the overlap of the hyperparameters selected across all external training folds. If the overlap was more than 80%, we considered the model to be stable and we trained the model on the complete dataset with the most frequently selected hyperparameter set. In this case, new data were being tested on the new complete model. If the overlap was between 60% and 80% we considered the model to be moderately unstable and we tested new data by averaging their predictions over all folds. If the overlap was less than 60% we considered the model to be unstable and we removed it from our set.

1.5 Post-hoc binarization

We have provided a post-hoc approach to analyze and visualize binary projection patterns in the mouse brain, primarily for facilitating comparison to previous studies (Ji et al., 2014). The binarization threshold was found by maximizing the area under the “receiver operating characteristic (ROC)” curve (auROC) value (Fawcett, 2006). In an ROC analysis, classification scores are converted to binary patterns based on a threshold and the accuracy score between measured and predicted binary patterns is estimated as the ratio between the true positive rate (TPR) and the false positive rate (FPR) (eq. 3).

$$\begin{aligned} TPR &= \frac{TP}{P} = \frac{\text{Positives classified correctly}}{\text{Total number of Positives}} \\ FPR &= \frac{FP}{N} = \frac{\text{Negatives classified incorrectly}}{\text{Total number of Negatives}} \\ ROC_{score} &= \frac{TPR}{FPR} \end{aligned} \quad (3)$$

Positives and Negatives correspond to the samples from the positive and negative class, respectively, which in our case correspond to the presence and absence of strong projections from a source area, respectively.
The strength of ROC analysis lies in the application of approximately all possible thresholds in the range 0-1, leading to a curve of approximately all possible TPR and FPR values. The auROC is estimated as the integral of the area under the curve that

represents the potential quality of classification performance and reveals the optimal threshold as the point on the curve furthest away from a 45 degree line: the higher the curve from the line, the better the performance (Fawcett, 2006).
In order to apply ROC analysis on our continuous data, predicted patterns have to be converted to classification scores and an external threshold is needed to convert the measured projection patterns to binary ones. This is achieved by setting up an external threshold set, different from the internal one used in ROC analysis. Moreover, the predicted patterns are transformed to classification scores with the standard logistic sigmoid function: $f(x) = \frac{1}{1+e^{(-x)}}$
Therefore, for each external threshold in the set, the optimal auROC is estimated as the output of ROC analysis between the measured patterns binarized from the threshold and the predicted patterns that are converted to scores. The selected threshold is the one with the maximum optimal auROC value. We refer to this in figure 3g in the main text as multi-ROC curves.

1.6 Gene Enrichment Analysis

We used gene ontology (GO) enrichment analysis to investigate the biological relevance of groups of genes found to be predictive of connectivity (Rivals et al., 2007). The hypergeometric test (Rice, 2007), was applied for estimating the statistical significance of the number of genes with a particular annotation being amongst the most predictive genes in our procedure, relative to the occurrence of genes with this annotation in similarly sized groups drawn randomly from the entire gene set (Rivals et al., 2007).
We applied GO enrichment analysis as follows. First, we created for each experiment a group of strong gene predictors by selecting genes with coefficients exceeding the 99th percentile (Results section, subsections 3.1 and 3.2), and we also selected genes with high coefficient scores in the spatial gene modules of interest (Results section, subsection 3.3). Second, the hypergeometric test was applied to each selected gene group. Third, annotations for which the hypergeometric test returned a p-value lower than 0.05, were considered significant and were collected in a table.
The ontology annotations and the gene set for the randomly drawn subsets were taken from the *org.Mm.eg.db* database (table 1) that contains genome-wide annotations for the mouse species.

2 Use Cases

This section describes a number of use cases from which potential users of the workflow can benefit.

2.1 Creation of a regionalized connectivity array based on layer-specific projection patterns

Our predictive workflow has incorporated regionalized connectivity models provided by the MCM tool (Knox et al., 2018). Specifically, we applied the MCM tool to measured projection patterns aggregated across all cre-lines. The output was a regionalized connectivity array between anatomical brain areas, for which both source and target cortical areas were layer-specific.

We investigated the differences in projection patterns across source areas derived from cre-lines wherein viral injections labeled cells according to different laminar profiles. Figure S1 shows an indicative subset of the regionalized array as well as a similarity matrix between source cortical areas with different laminar profiles. The similarity matrix was created by estimating the Spearman's rank coefficient (also referred to as rho) between the different source areas. We clustered the source areas on the similarity matrix based on their laminar profile. In addition, we applied the silhouette score for quantifying the clustering quality, which is a standard measure in clustering analysis with values ranging from -1 to 1 and reflecting the cluster cohesion (Rousseeuw, 1987). The silhouette score was 0.61, which was considered to reflect the cohesive clusters found in figure S1. Therefore, we regarded projection patterns of source areas with cells labeled according to the same laminar profile to be more similar compared to those of different profiles. This observation might be in line with the findings of (Harris et al., 2019), according to which long-range intracortical and corticothalamic projections from different laminar profiles follow distinct connectivity rules.

In order to estimate the significance of that finding, we generated surrogate clusters by randomly distributing the projection densities across source areas 1000 times. In addition, we estimated a p-value based on the number of times that the silhouette score of the surrogate clusters was greater than the score of the actual clusters. The resulting p-value was 0, which indicated that differences in projection patterns from source areas with different laminar profiles were significant.

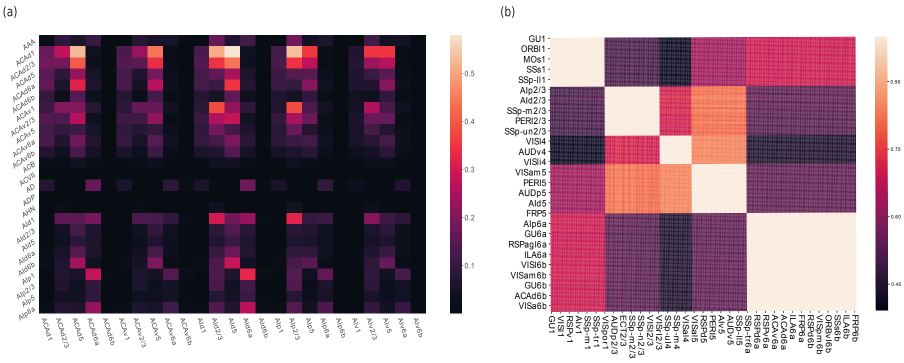

Fig. 1: Heatmaps of a layer-specific regionalized connectivity array. (a) Subset of the array comprised of a selected set of 25 target and 25 source brain areas. x-axis: source brain areas. y-axis: target brain areas. (b) Similarity matrix of source brain areas which are clustered based on their laminar profiles. Both axes correspond to clustered laminar profiles of source brain areas. The similarity matrix was created by taking all pairs of source areas and estimating the Spearman's rho between their projection patterns. All distinct blocks with values greater than 0.9 represent pairs of areas with the same profile. This suggests that groups of areas with the same laminar profile have more similar projection patterns compared to groups of areas with different profiles.

2.2 Link to Mouse Connectivity Models

We linked our predictive workflow to the Mouse Connectivity Models (MCM) tool provided by the Allen Institute for Brain Science (Knox et al., 2018). The MCM tool comprises a set of procedures, based on penalized regression, for constructing connectivity matrices on a volumetric scale of 100 μm^3 or on a regionalized scale of structural brain areas (see supplementary file 1). This tool enabled us to integrate the 1397 tract tracing experiments into one connectivity matrix and analyze the differences in projection patterns from different laminar profiles.
Besides integrating our analysis with the Mouse Connectivity Models (MCM) tool (Knox et al., 2018), we implemented and documented use cases that are related to the tool. Specifically, the users can download data with the MCM tool and then obtain the array of interest based on volumetric or regionalized preference. Alternatively, users can give their own projection patterns as input, for instance based on reconstructed axonal projections or predicted ones generated from our models. Such input will be mapped automatically to the volumetric scale of 100 μm^3 and then provided to the MCM tool for connectivity array construction in a volumetric or regionalized form.

2.3 Incorporating new user data

The capability of the developed workflow to incorporate external or user-generated data was validated in the form of use cases. A user can get predictions for a new gene expression dataset by selecting a layer and class profile (i.e. L5 CT corresponding to layer 5 corticothalamic specific projections) and a source area of interest (i.e. MOp or primary motor area). The model most closely associated with the selected preferences

will automatically be selected and the predicted axonal projections will be returned. Moreover, a user can decide whether to trust the selected model based on its performance score (r^2) or select another model. Furthermore, the patterns can be converted to a laminar specific regionalized connectivity matrix based on the MCM tool as described in subsection S2.2. Finally, the visualization part of our workflow can be used for a visual inspection of the various projection patterns (subsection S2.4).

2.4 Brain Visualization

Part of our predictive worfklow comprised visualizations of brain volumetric data in the form of cortical flatmaps and brain slices. We constructed both cortical flatmaps oriented along the anterior-posterior and left-right axes, and brain slices oriented along the inferior-superior and left-right axes. The data were then converted to either JavaScript Object Notation (JSON) format or in Neuroimaging Informatics Technology Initiative (NIfTI) format, in order to be visualized through an API call to the Scalable Brain Atlas (SBA) Composer, a 3D brain visualization tool (Bakker et al., 2015). The SBA Composer provides 3-dimensional visualizations of brain volumes through a user-friendly interface.

In order to appreciate the spatial context of our predicted data, they were mapped to a 25 or 10 μm^3 volumetric scale based on annotation volumes. For the cortical flatmaps, we mapped the input data to the 10 μm^3 volumetric scale based on the Allen annotation volume of the same resolution. We then used the MCM tool to map the 3D volume to a 10 μm^3 cortical flatmap template and to average the cortical values over all layers. The flatmap template was downloaded from the informatics archive of the Allen Institute (Main table 1). For the brain slices, we mapped the input data to the 25 μm^3 volumetric scale based on the Allen annotation volume of the same resolution. We then selected a point across the anterior-posterior axis in order to create a 2D slice at the coordinates of interest.

The resulting cortical flatmaps and brain slices were plotted overlaid with the templates that were provided together with the annotation volumes by the Allen Institute (Main table 1), hence comprising a set of components. To achieve this we used the red, green and blue channels to represent the components as images and then mixed them by selecting per pixel the component with the highest intensity. We repeated this procedure over all pixels of the input images and then we plotted the figures using standard Python libraries (see Main subsection 2.1). More details about the aforementioned formats and tools can be found in Main table 1.

3 Supplementary Figures

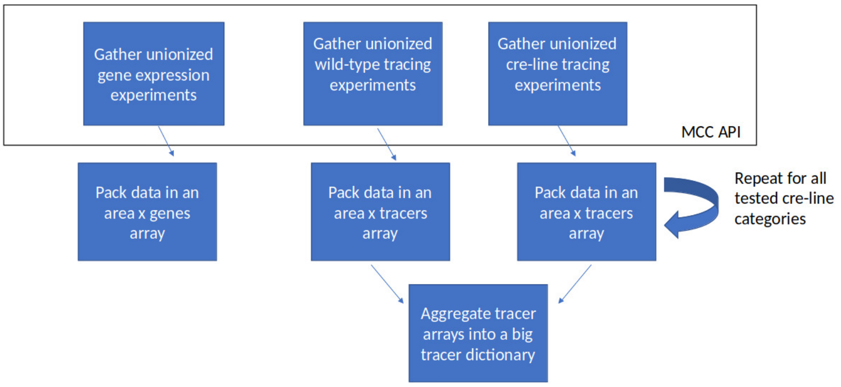

Fig. 2: Flowchart describing the data retrieval pipeline.

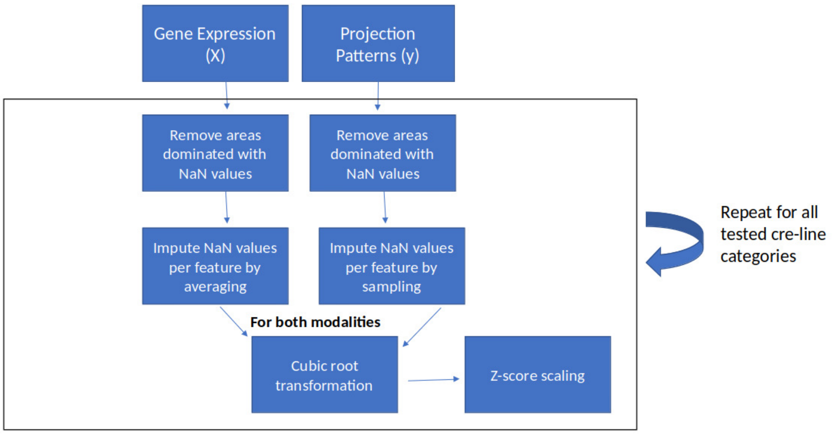

Fig. 3: Flowchart describing the data preprocessing pipeline.

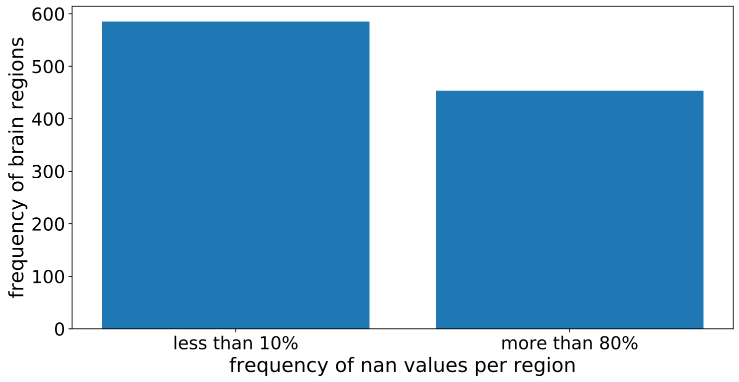

Fig. 4: Histogram with frequency of NaN value occurrence per brain area. Gene Expression dataset. The total number of brain areas is 1038, as defined by the Allen CCF v3.0

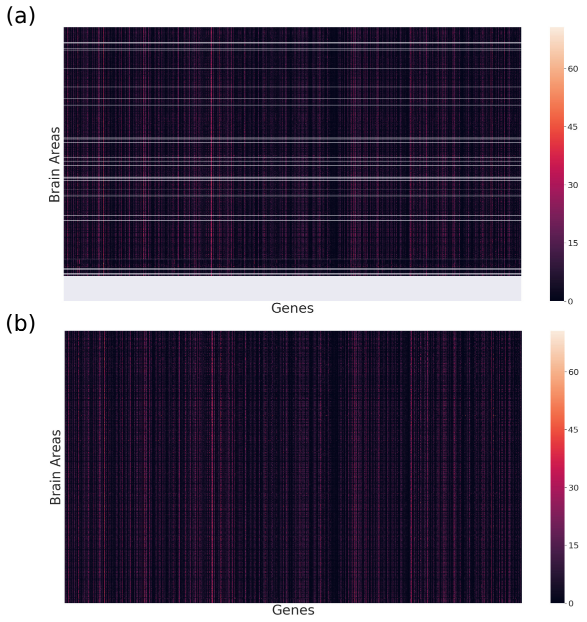

Fig. 5: Gene Expression dataset before removing brain areas dominated by NaN values (a) and after their removal (b). The NaN values are represented by the gray color.

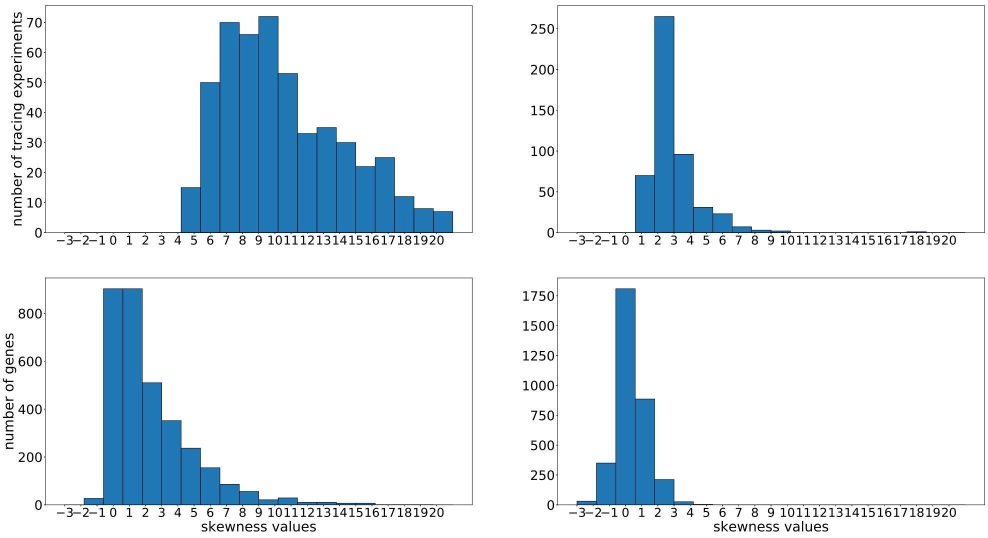

Fig. 6: Skewness distributions for the wild-type projection volume dataset (top) and the gene expression datasets (bottom) before pre-processing (left panels) and after pre-processing (right panels).

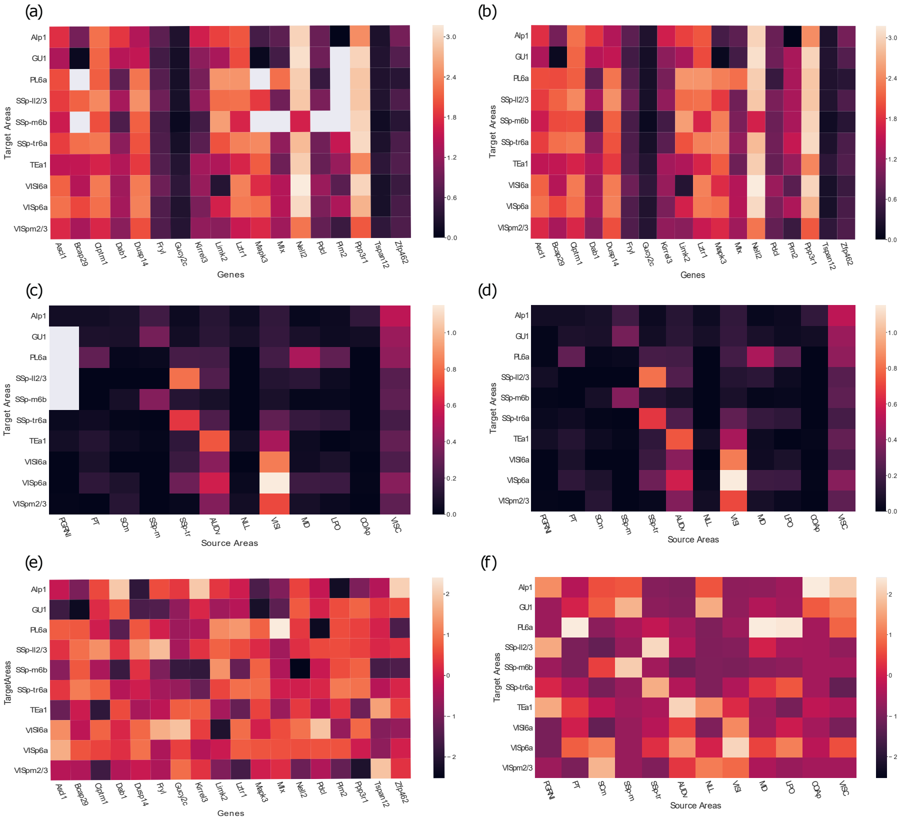

Fig. 7: The gene expression and wild-type axonal projection datasets during the different pre-processing steps. (a-b) Subset of the Gene Expression dataset containing sparse NaN values (a) and the same subset after the median imputation (b). (c-d) Subset of the wild-type projection dataset containing sparse NaN values (c) and the same subset after the sampling imputation (d). (e-f) Z-score transformation of the gene expression dataset (e) and the wild-type projection volume dataset (f). The NaN values are shown in gray. The non-NaN values have been cube-root transformed for clarity. In (a-d) the brain areas were chosen to obtain examples with some NaN values present and examples without any NaN values.

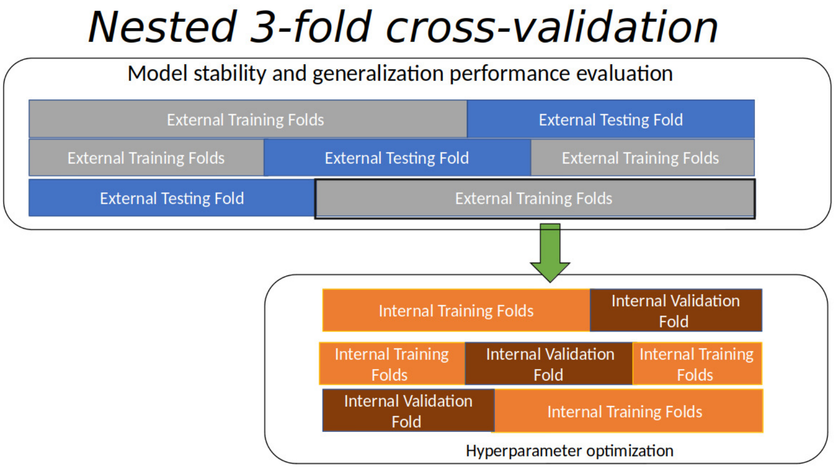

Fig. 8: Schematic describing the structure of the nested cross-validation method.

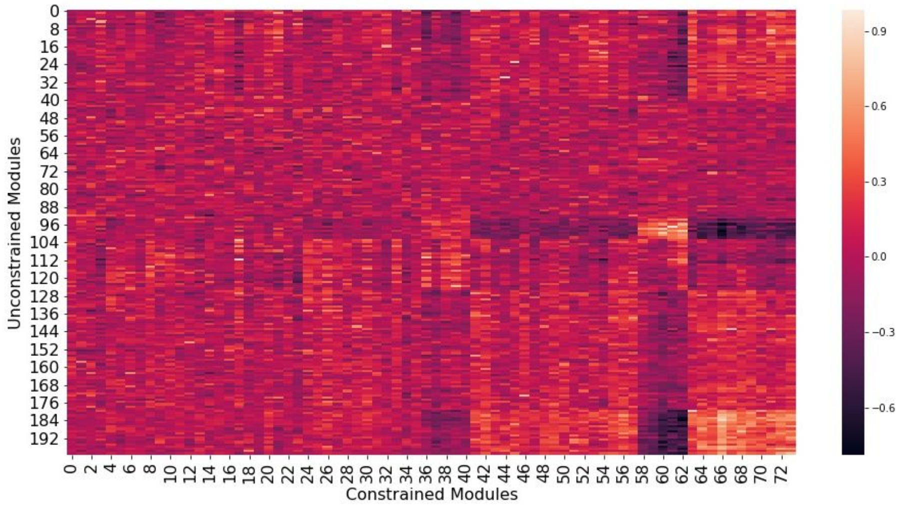

Fig. 9: Heatmap displaying correlations between the constrained and unconstrained spatial gene modules. x-axis: constrained modules. y-axis: unconstrained modules. The correlations were estimated with the Spearman's rank correlation coefficient. 58% out of 6600 correlations in total were considered significant ($p \leq 0.05$). However, there are no evident correlation patterns between the two types of modules.

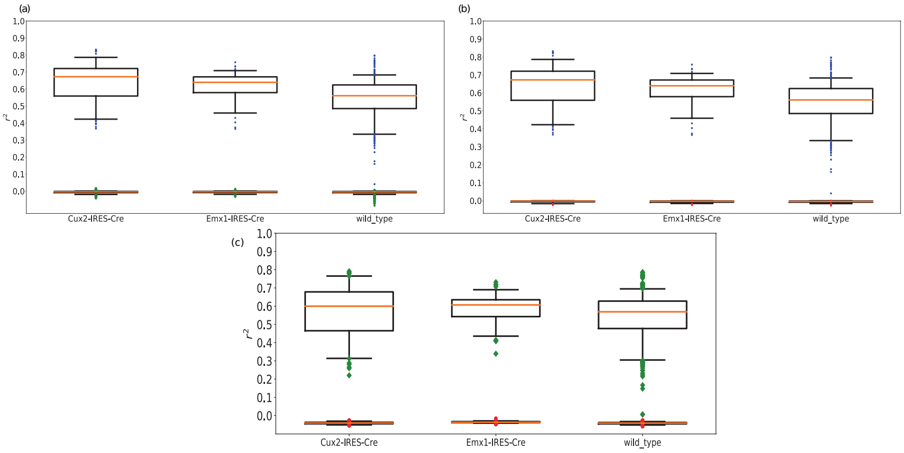

Fig. 10: Comparison of prediction performance between models trained using the actual data and null or surrogate models. (a) Comparison of surrogate (bottom panel) and actual (top panel) models trained to predict a number of tract tracing datasets using gene expression data. x-axis in this panel and the other two panels: datasets - Cux2-IRES-Cre (left), Emx1-IRES-Cre (middle), wild-type (right). y-axis: r^2 scores. The red line and box are as in (b) while the blue and green dots are outliers for the regression of the actual and surrogate data respectively. (b) Comparison of null (bottom panel) and actual (top panel) models trained to predict a number of tract tracing datasets using gene expression data. The color conventions are as in panel (b). (c) Comparison of surrogate (bottom panel) and actual (top panel) models trained to predict a number of tract tracing datasets using spatial gene modules.

References

Bakker, R., Tiesinga, P., and Kötter, R. (2015). The scalable brain atlas: Instant web-based access to public brain atlases and related content. *Neuroinformatics*, 13(3):353:366.

Bishop, C. M. (2006). *Pattern Recognition and Machine Learning*. Information Science and Statistics. Springer, first edition.

Breiman, L. (2001). Random forests. *Machine Learning*, 45(1):5–32.

Dietterich, T. G. (2000). Ensemble methods in machine learning. In *Proceedings of the First International Workshop on Multiple Classifier Systems*, pages 1–15.

Fawcett, T. (2006). An introduction to roc analysis. *Pattern Recognition Letter*, 27:861–874.

Friedman, J., Hastie, T., and Tibshirani, R. (2009). *The Elements of Statistical Learning. Data Mining, Inference, and Prediction*. Springer Series in Statistics. Springer, 2 edition.

Harris, J. A. et al. (2019). Hierarchical organization of cortical and thalamic connectivity. *Nature*, 575:195–202.

Ji, S., Fakhry, A., and Deng, H. (2014). Integrative analysis of the connectivity and gene expression atlases in the mouse brain. *Neuroimage*, 84:245–253.

Knox, J. E., Harris, K. D., Graddis, N., and Whitesell, J. D. (2018). High resolution data-driven model of the mouse connectome. network neuroscience. *Neuroscience*, 3(1):217–236.

Kohavi, R. (1995). A study of cross-validation and bootstrap for accuracy estimation and model selection. In *Proceedings of the 14th international joint conference on Artificial intelligence (IJCAI*, volume 2, pages 1137–1143.

Rice, J. A. (2007). *Mathematical Statistics and Data Analysis*. Mathematics of Computation. Duxbury Press, 3 edition.

Rivals, I., Personnaz, L., Taing, L., and Potier, M. C. (2007). Enrichment or depletion of a go category within a class of genes: which test? *Bioinformatics*, 23(4):401–407.

Rousseeuw, P. (1987). Silhouettes: a graphical aid to the interpretation and validation of cluster analysis. *Journal of Computational and Applied Mathematics*, 20:53–65.

Tikhonov, A. N. and Arsenin, V. Y. (1977). *Solution of Ill-posed Problems*. Mathematics of Computation. Winston & Sons, 1 edition.

Varma, S. and Simon, R. (2006). Bias in error estimation when using cross-validation for model selection. *BMC Bioinformatics*, 7:91.
